# Supplementary material for: Unveiling the significance of women’s role in health-seeking behavior during suspected malaria fever in risk populations of Nepal: Mixed methods cross-sectional study
Source: PLOS Glob Public Health. 2024 Sep 4;4(9):e0003697. doi: 10.1371/journal.pgph.0003697 (PMC11373845; doi:10.1371/journal.pgph.0003697)
Supplement: Attachment — Submitted filename: response to reviewer.doc [file pgph.0003697.s002.doc]

Dear Reviewer,

Good evening.

Thank you very much for your valuable inputs and suggestions. Based on your comments, we have made the following revisions and finalized both the marked-up copy (revised manuscript with track changes_PGPH-D-24-00163) and the unmarked version (manuscript_PGPH-D-24-00163):

**In the Introduction Section:**

- In the first paragraph, the word 'Plasmodium' has been italicized as per your suggestion.
- The third sentence of the first paragraph has been replaced with information from the World Malaria Report 2023 of the World Health Organization: "In 2022, global malaria cases surged to 249 million, while incidence rates decreased from 81 cases per 1000 individuals at risk in 2000 to 57 cases per 1000 in 2019. Despite a slight 3% increase in 2020, incidence rates have remained consistent over the past three years. Specifically, in 2022, the incidence of malaria cases was recorded at 58 per 1000 individuals at risk" [5]. The reference has been updated accordingly.
- As per your suggestion, the last sentence of the second paragraph has been added: "In Nepal, confirmed malaria cases increased from 491 in Fiscal Year 2022/23 to 533 in Fiscal Year 2023/24, predominantly due to imported cases (95.6%). The incidence of Plasmodium falciparum infections also escalated, representing over 28.33% of all cases in Fiscal Year 2023/24. Among Nepal's total confirmed malaria cases, Kanchanpur district reported 28 cases in Fiscal Year 2023/2024" [7].
- The citation number in the first sentence of the third paragraph has been corrected to [8-10] as per your comment.
- Similarly, the citation number in the second sentence of the third paragraph has been corrected to [11] based on your feedback.

**In the Results Section:**

- The word 'Large' has been deleted and restructured as 'The overwhelming majority of respondents were Hindu (89.4%)' as per your suggestion.

**In the Discussion Section:**

- Citation numbers of references 9 and 10 have been replaced with 12 and 13, respectively. Similarly, subsequent citations have been corrected accordingly.

**In the References:**

- Reference number 5 has been edited as follows:

5. World Health Organization. World Malaria Report 2023. Geneva, Switzerland; 2023. Accessed on: 2024/4/7. Available at: <https://www.who.int/teams/global-malaria-programme/reports/world-malaria-report-2023>

- New reference number 7 has been added:

7. Department of Health Services/Ministry of Health and Population of Nepal. Annual Health Report 2079/2080. Kathmandu, Nepal; 2024. Accessed on: 2024/4/7. Available at: <https://dohs.gov.np/annual-health-report-2079-80/>

- New reference numbers 9 and 10 have been also added as per your suggestion;

9. Hankin JR, Wright ER. Reflections on fifty years of medical sociology. J Health Soc Behav. 2010;51 Suppl:S10-S14. Accessed on: 2024/4/7. Available at: <https://doi.org/10.1177/0022146510383840>

10.	Merson MH, Black RE, Mills AJ, editors. International public health programs, systems and policy. Barb House, London W67PA UK/Massachusetts USA: Jones & Bartlett Publishers International; 2001. pp. 53-75.

- All other reference numbers (8, 11-28) have been revisited, edited, and corrected accordingly.

Once again, thank you for your valuable comments and cooperation.

With regards,

Ashok Kumar Paudel (Corresponding Author)

Email: [ashokkpaudel@gmail.com](mailto:ashokkpaudel@gmail.com)
