# Supplementary material for: Unveiling the significance of women’s role in health-seeking behavior during suspected malaria fever in risk populations of Nepal: Mixed methods cross-sectional study
Source: PLOS Glob Public Health. 2024 Sep 4;4(9):e0003697. doi: 10.1371/journal.pgph.0003697 (PMC11373845; doi:10.1371/journal.pgph.0003697)
Supplement: Attachment — Submitted filename: response to reviewer_june14.doc [file pgph.0003697.s003.doc]

Dear Reviewer,

Good evening.

Thank you very much for your valuable inputs and suggestions. Based on your comments, we have made the following revisions and finalized both "Marked-up copy" and "Revised Manuscript with Track Changes" manuscripts.

**In the Introduction Section:**

- As per your suggestion, based on the World Malaria Report 2023 by WHO, the malaria incidence rate has been reviewed, corrected, and included in the third sentence of the first paragraph in the Introduction section.

Previous sentence "In 2022, global malaria cases surged to 249 million, while incidence rates decreased from 81 cases per 1000 individuals at risk in 2000 to 57 cases per 1000 in 2019. Despite a slight 3% increase in 2020, incidence rates have remained consistent over the past three years. Specifically, in 2022, the incidence of malaria cases was recorded at 58 per 1000 individuals at risk" **has been deleted and written as** " Globally, although there was a positive decline in malaria incidence rates from 81 cases per 1,000 people at risk in 2000 to 57 cases per 1,000 in 2019, the COVID-19 pandemic significantly impacted progress. In 2022, the number of global malaria cases surged to 249 million, exceeding pre-pandemic levels. This rise indicates a concerning increase in incidence rates, despite only a slight increase observed in 2020"[5]

- As per given valuable suggestions, within the context of the research objective, more information about healthcare-seeking behavior has been included in the third paragraph of the Introduction section.

In third paragraph of the introduction section following second and third sentences has been added as;

"Any activities under taken by individuals, who perceive them to have a health problem or to be ill for the purpose of finding appropriate remedy is health seeking behavior [11-13]. It is influenced by a large number of individual and social factors; is a complex outcome of many factors operating at individual, family and community level [14]."

- The citation number 11-14 has been added and subsequent citation numbers has been corrected.

**In the Discussion Section:**

- More explanation has been added to the third paragraph of the Discussion section regarding beliefs related to women's bed net usage during menstruation. This pertains to the results on cultural beliefs and women's bed net usage during menstruation.

In third paragraph, third sentence has been added as " This result indicates that cultural beliefs about bed net use during menstruation significantly influence healthcare seeking behavior. Specifically, individuals who believe that women can use bed nets during menstruation are almost twice as likely to opt for modern health facilities when seeking healthcare. The statistical analysis supports this finding with a p-value of 0.016, which is less than the commonly used threshold of 0.05, indicating that the association is statistically significant."

- Similarly, subsequent citations have been corrected accordingly.

**In the References:**

- Reference number 11-14 has been added as below:

11. Kasl SV, Cobb S. Health behavior, illness behavior, and sick-role behavior. II. Sick-role behavior. Arch Environ Health. 1966;12(4):531-541. Accessed on: 2024/6/12. Available at: <https://doi.org/10.1080/00039896.1966.10664421>
12. Ward H, Mertens TE, Thomas C. Health seeking behaviour and the control of sexually transmitted disease. Health Policy Plan. 1997;12(1):19-28. Accessed on: 2024/6/13. Available at: <https://doi.org/10.1093/heapol/12.1.19>
13. Uzochukwu BSC, Onwujekwe OE. Socio-economic differences and health seeking behavior for the diagnosis and treatment of malaria: A case study of four local government areas operating the Bamako initiative program in southeast Nigeria. Int J Equity Health. 2004;3(6). Accessed on: 2024/6/13. Available at: <https://doi.org/10.1186%2F1475-9276-3-6>
14. Jain M, Nandan D, Misra SK. Qualitative assessment of health-seeking behavior and perceptions regarding quality of health care services among rural communities in District Agra. Indian J Community Med. 2006;31:140-144. Accessed on: 2024/6/13. Available at: <https://www.scirp.org/reference/referencespapers?referenceid=811307>

- All other reference numbers have been subsequently revisited, edited, and corrected.

**Regarding to data availability statement-** Corrected as "All data underlying the findings described in this manuscript will be freely available to other researchers".

Once again, thank you for your valuable comments and cooperation.

With regards,

Ashok Kumar Paudel (Corresponding Author)

Email: [ashokkpaudel@gmail.com](mailto:ashokkpaudel@gmail.com)
